# Myxoid Liposarcoma: Treatment Outcomes from Chemotherapy and Radiation Therapy

**DOI:** 10.1155/2018/8029157

**Published:** 2018-11-01

**Authors:** Varun Chowdhry, Saveli Goldberg, Thomas F. DeLaney, Gregory M. Cote, Ivan Chebib, Jason Kim, Santiago A. Lozano-Calderón, Karen De Amorim Bernstein

**Affiliations:** ^1^Department of Radiation Medicine, Roswell Park Comprehensive Cancer Center, Buffalo, NY, USA; ^2^Department of Radiation Oncology, Massachusetts General Hospital, Boston, MA 02114, USA; ^3^Division of Hematology/Oncology, Massachusetts General Hospital, Boston, MA 02114, USA; ^4^Department of Pathology, Massachusetts General Hospital, Boston, MA 02114, USA; ^5^Department of Orthopedic Oncology, Massachusetts General Hospital, Boston, MA 02114, USA

## Abstract

**Introduction:**

Myxoid liposarcoma (MLS) is a subtype of liposarcoma characterized morphologically by lipomatous differentiation with a myxoid stroma. The purpose of this study was to review clinical and pathological information for patients treated for MLS at our institution to better understand neoadjuvant and adjuvant therapy.

**Materials and Methods:**

An institutional database of sarcomas was queried for patients who were treated for MLS at our institution between 1992 and 2013. Survival curves were constructed using Kaplan–Meier analysis, and univariate and multivariate statistics were performed using the Cox-proportional hazards model and using linear regression.

**Results:**

A total of 85 patients with myxoid liposarcoma were identified. The mean and median histologic response rate to treatment for patients who received preoperative radiation therapy was 77.6%. Five-year disease-free survival, distant metastasis-free survival, local recurrence-free survival, and overall survival were 78.6% (95% CI: 67.8–86.1), 84.7% (95% CI: 74.5–91.0), 95.6% (95% CI: 86.9–98.6), and 87.5% (95% CI: 77.2–93.3) respectively. On univariate analysis, there was a trend towards higher necrosis or treatment response rates in patients who received concurrent chemotherapy, 84.7% (95% CI: 75.9–93.4) and 69.5% (95% CI: 55.1–83.8), *p*=0.061. Tumor size was associated with inferior disease-free and overall survival. Hazard ratio for disease-free survival is 1.08 (per cm) (95% CI: 1.01–1.16), *p*=0.019.

**Conclusions:**

Myxoid liposarcoma exhibits histological response to chemotherapy and radiation therapy. Tumor size appears to be greatest predictor of long-term disease control and overall survival. We were not able to show that chemotherapy provides a clinical benefit with regard to local control, disease-free survival, or overall survival. However, it is important to note that the selected usage of chemotherapy in the highest risk patients confounds this analysis. Further investigation is needed to help better determine the optimal use of chemotherapy in this group of patients.

## 1. Introduction

Myxoid liposarcoma (MLS) is a subtype of liposarcoma that represents a distinct pathological entity characterized morphologically by tumor cells within a myxoid stroma with a rich, branching thin-walled vasculature, and focal lipomatous differentiation. The MLS subtype represents approximately 1/3 of all liposarcomas and 10% of adult soft tissue sarcomas [1]. MLS is associated with chromosomal translocations consisting of the FUS and DDIT3 (CHOP) genes t(12; 16)(q13; p11) or the EWS and DDIT3 (CHOP) genes t(12; 22)(q13; q12) [2]. There is evidence to suggest that MLS is both radioresponsive and radiosensitive [3]. Marked reduction of tumor volume has been noted during treatment, with one series showing a median reduction in tumor volume from the start to end of treatment of 59% [4].

Comparative analysis of MLS versus other sarcoma subtypes suggests greater response rates in MLS with the addition of anthracycline-based chemotherapy [5]. In one series, there is a suggestion that doxorubicin and ifosfamide can result in favorable long-term outcomes [6]. However, there is relatively limited information on factors that predict for overall outcome in MLS, particularly with regard to the benefits of combined trimodality therapy. The purpose of this single institution MLS series is to evaluate clinical variables that may predict for improved outcomes and thereby help guide management or future clinical trials.

## 2. Materials and Methods

After IRB approval, our oncology registry database was queried for patients who were treated for MLS at our institution between 1992 and 2013. Only patients with no evidence of metastatic disease treated with definitive limb-salvage therapy were included in this series. All patients had pathological confirmation by a sarcoma-specialized pathologist either through secondary review of slides from another institution or through direct pathological sampling obtained at our institution. All patients were evaluated in a multidisciplinary care setting for indications and suitability to receive preoperative chemotherapy and radiation therapy. Patients treated with definitive therapy for their disease were included in this analysis. Sixty-nine out of 85 patients (81%) had the initial biopsy performed at our institution, and seventy-nine patients (93%) had definitive surgical resection performed at our institution. Patients diagnosed with MLS, including high-grade MLS (formerly myxoid/round cell liposarcoma), were included in this analysis. Demographic, clinical, radiographic, pathologic, and treatment outcomes were captured. Statistical analysis using both univariate and multivariate models were conducted using SAS software (SAS version 9.4; 100 SAS Campus Drive, Cary, NC 27513). Survival curves were constructed using Kaplan–Meier analysis, and univariate and multivariate statistics were performed using the Cox-proportional hazards model and using linear regression.

## 3. Results

Demographic information for patients in this series is listed in Table 1. Seventy-three of 85 patients had sarcomas of the lower extremity (85.9%), with complete breakdown by location shown in Table 1. The median follow-up for patients in this series was 85.2 months (range, 4–250 months). Sixty-seven (78.8%) patients were treated with preoperative radiotherapy, and 15 (17.6%) patients were treated with postoperative radiation therapy, generally due to close or positive postoperative margins. Ten patients (11.8%) in this series did not receive any radiotherapy. Seven patients (8.2%) were treated with both pre- and postoperative radiation therapy. Patients were treated with either 2D radiotherapy prior to 2000, 3D conformal radiation therapy (3D-CRT), or intensity-modulated radiotherapy (IMRT). Thirty-nine (45.9%) patients were treated with preoperative chemotherapy, and 22 (25.9%) of these patients received at least one cycle of postoperative chemotherapy. The chemotherapy regimens were almost entirely anthracycline-based, including mesna, doxorubicin, ifosfamide, and dacarbazine (MAID) or doxorubicin, ifosfamide, and mesna (AIM) chemotherapy, with the exception of one patient who received bevacizumab alone on a clinical trial.

**Figure 1:**
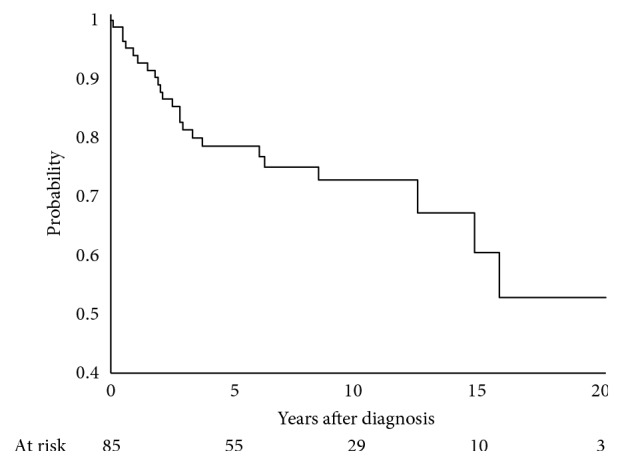
Disease-free survival at 5 years, 78.6% (95% CI: 67.8–86.1).

**Figure 2:**
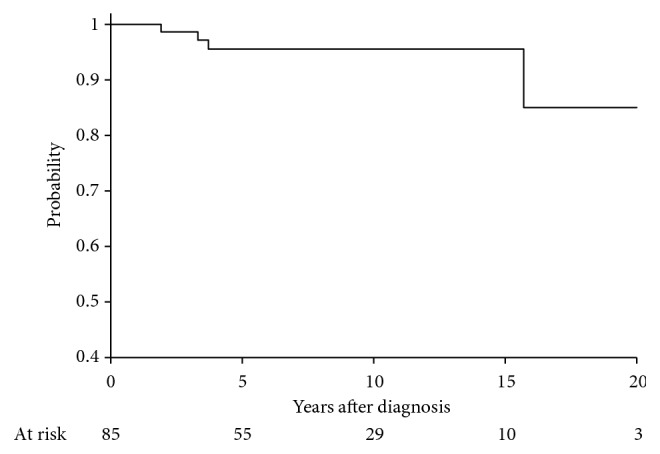
Local control at 5 years, 95.6% (86.9–98.6).

**Figure 3:**
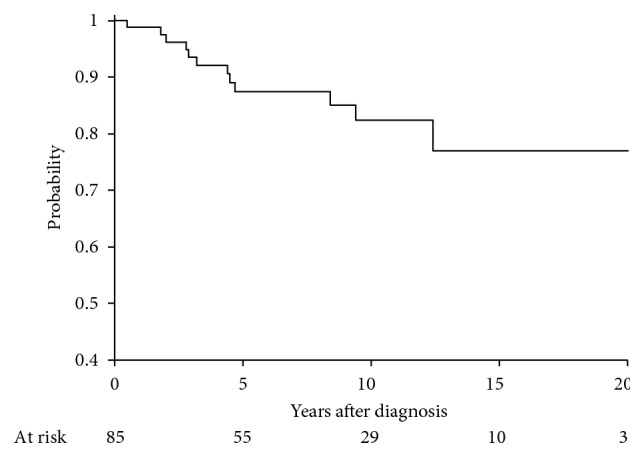
Overall survival at 5 years, 87.5% (77.2–93.3).

**Figure 4:**
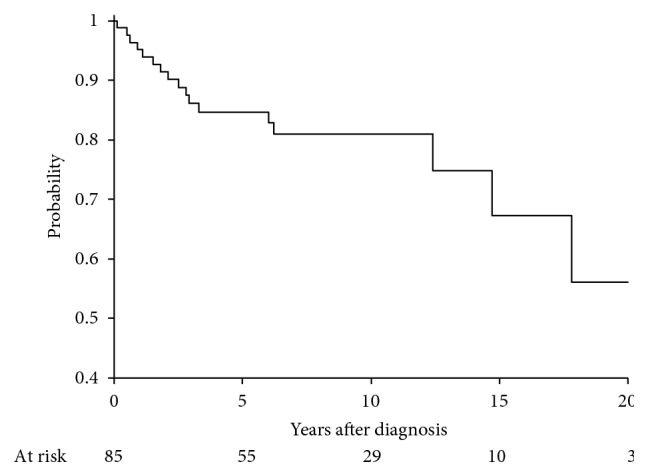
Distant metastases-free survival at 5 years, 84.7 (74.5–91.0).

Five-year disease-free survival, distant metastasis-free survival, local control, and overall survival were 78.6% (95% CI: 67.8–86.1), 84.7% (95% CI: 74.5–91.0), 95.6% (95% CI: 86.9–98.6), and 87.5% (95% CI: 77.2–93.3), respectively (Figures 1–4). Four out of 85 (4.7%) patients experienced a local recurrence. Of these four patients, two had received preoperative radiotherapy. One patient received a preoperative dose of 4400 cGy, while the other patient received a preoperative dose of 3000 cGy. Out of these, 1 patient (25%) had received postoperative radiotherapy. This patient had not received preoperative radiotherapy.

Radiotherapy with or without chemotherapy was associated with a high degree of tumor response. The median percent necrosis for patients who received preoperative radiation therapy was 95% (range, 0–100%), with a mean necrosis rate of 77.6% (Table 2). Percent necrosis is a direct indicator of pathological response and was inversely associated with histologically intact residual tumor (e.g., 100% necrosis indicates that no histological tumor was left behind). Ten out of 85 patients had a pathological complete response (11.7%). On univariate analysis, there was a trend towards higher necrosis rates in patients who received concurrent chemotherapy, 84.7% (95% CI: 75.9–93.4) and 69.5% (95% CI: 55.1–83.8), *p*=0.061, compared with patients who did not receive concurrent chemotherapy. On multivariate analysis, the use of chemotherapy was significantly associated with increased rates of necrosis (Table 3). However, the addition of chemotherapy did not appear to be associated with improvements in disease-free or overall survival. In patients with tumor size ≥5 cm who had chemotherapy, OS: HR (hazard ratio) is 1.69 (95% CI: 0.54–5.25), *p*=0.367 and DFS: HR is 1.85 (95% CI: 0.81–4.19), *p*=0.143. Tumor size of <5 cm, 5–10 cm, and >10 cm has been associated with prognosis in sarcoma subtypes [7].

The use of radiation therapy with or without chemotherapy preoperatively resulted in overall very low rates of local recurrence (4.7% in all patients with 2/4 failures occurring in patients with negative margins and 2/4 failures occurring in patients with close (<1 mm) or positive margins).

With regard to pretreatment variables that could predict outcome, large tumor size, as analyzed by a continuous variable, was associated with inferior disease-free survival and local control. Large tumor size and Grade ≥ 2 were associated with inferior overall survival (Tables 4–7).

Short- and long-term toxicity was scored based on retrospective chart review and the RTOG acute and late toxicity scale [8]. Acute toxicity data were available for a total of eighty-three patients. Seventeen out of 83 patients (20%) experienced an RTOG acute skin toxicity Grade 3 or higher. Of these 17 patients, 9 had received chemotherapy (52.9%), while 8 patients did not receive chemotherapy (47.1%). These differences were not statistically significant (*p*=0.771). Late toxicity data were available for a total of 83 patients. Nine patients (10.8%) experienced an RTOG Grade 3 or higher late skin toxicity.

## 4. Discussion

In this series of 85 patients, we found a mean necrosis rate of over 77% in patients treated with preoperative radiation therapy. These data support the findings from other published series and clinical experience that suggest that MLS is a radiosensitive malignancy, as reflected by histological response. While it is not certain how histological response correlates with treatment outcomes, there is a suggestion from some published series that high levels of necrosis may correlate with improved outcomes [9, 10]. Additionally, the results of our series show a trend that the addition of concurrent chemotherapy increased necrosis to a mean of 82.3% as compared with 58.8% patients who were not treated with chemotherapy. Our study findings are also consistent with other sarcoma studies in which large tumor size is associated with inferior disease-free survival [11].

The high degree of response supports the use of preoperative therapy of these MLS patients.

While it is our practice to consider additional postoperative boost radiation therapy in patients with positive margins after preoperative radiation, the low rates of local failures make it difficult to perform more detailed analysis of related factors that may impact local control and survival. One of the local failures occurred in a patient who had a grossly positive margin and still had a local failure after postoperative radiation therapy.

The results of our series are concordant with other published series in MLS suggesting chemotherapy sensitivity [12]. Furthermore, our series is hypothesis generating in which chemotherapy may enhance the effect of radiotherapy. In our study, chemoradiotherapy was associated with histological response; we did not see a benefit of chemotherapy with disease-free or overall survival.

While patients in this retrospective series received different chemotherapeutic agents, a large randomized study did not show a benefit to histologically tailored chemotherapy, over a standard regimen [13]. Given the low rate of local failures overall, the number of events make statistical analysis about factors that either enhance locoregional control difficult. From the literature, there is uncertainty whether high necrosis rates correlate with improved outcome. Some reports [14, 15] have shown no overall clinical benefit with high tumor necrosis rates, while another has suggested that pathological necrosis does correlate with clinical outcome [16]. Although our series did not demonstrate a definite correlation of the degree of necrosis with disease-free survival or overall survival, it is important to recognize that selection bias likely led to larger, higher risk tumors receiving chemotherapy (which was then associated with greater pathologic necrosis), thus confounding these data. Furthermore, it is possible that mechanisms behind tumor necrosis could vary and that patients with tumor necrosis as a result of a rapidly dividing tumor outgrowing its blood supply could have a different outcome compared with a patient who develops a necrotic tumor due to a significant response. The rarity of this tumor, however, would make a randomized clinical trial designed to address the effectiveness of adjuvant chemotherapy specifically for MLS difficult to complete.

The findings of our study support the importance of multidisciplinary care in the management of patients with MLS. In addition to radiotherapy, chemotherapy also appears to enhance the histological response rates to treatment.

Due to the low numbers of local failures, more definitive conclusions on the impact of chemotherapy on local control in MLS cannot be made. The use of concurrent, neoadjuvant chemotherapy, with the potential to reduce the incidence of metastatic disease has a theoretical appeal because of the poor prognosis of patients who ultimately develop metastatic disease [17].

There are some limitations of study that warrant further discussion. First, as a single institution study, there is inherent selection and management bias. The retrospective nature of our series also limits our ability to draw conclusions regarding specific therapies.

Our series did not find any differences in toxicity in patients who received chemotherapy, although it is likely that chemotherapy adds at least some degree of toxicity in patients who are treated with radiation therapy that may not have been captured in a retrospective analysis. It is possible that chemotherapy may have resulted in higher rates of acute Grade 2 or higher skin toxicity; these may not have been captured due to the retrospective nature of our series. Another limitation of this series was that the patients reviewed performed where largely the population of patients was surveilled using CT imaging to detect distant recurrence. There is a recent report that whole-body MRI may be more sensitive at detecting a pulmonary metastases compared with CT, and it is possible that this is more sensitive with regard to the detection of metastatic disease [18]. Furthermore, it is possible that at least some of the patients in this series could have been found to have metastatic disease even prior to the initiation of definitive therapy.

While the addition of chemotherapy provides for a means of treatment intensification in patients with a high risk of local and distant recurrences, the results of our series also present an opportunity to evaluate the role of treatment deintensification. The high rates of local control with a median preoperative radiotherapy dose of 50 Gy suggest that it may be reasonable to de-escalate therapy in selected patients, which is the subject of an ongoing international clinical trial [19]. However, more investigation is required to better understand in which patients such treatment deintensification is safe.

## 5. Conclusions

The results of our study support the notion that myxoid liposarcoma has a high rate of histological response with combined chemotherapy and radiotherapy, with low rates of local failure with trimodality therapy. The high pathological response rates to chemotherapy are hypothesis generating. While some reports [16] have suggested that high necrosis rates may correlate with improved clinical outcomes, our study, while not specifically designed to address this issue, did not demonstrate a correlation between necrosis and clinical outcome. We were not able to show that chemotherapy provides a significant clinical benefit with regard to local control, disease-free survival, or overall survival, although the likely selected usage of chemotherapy in the highest risk patients confounds this analysis. We agree with the National Comprehensive Cancer Network (NCCN) guidelines that chemotherapy should be considered in patients with large, high-grade sarcomas [20]. Further research is required to understand which patients benefit the most from chemotherapy, and which patients may benefit from treatment deintensification.

## Figures and Tables

**Table 1:** Demographic information.

Total number of patients	85
Male	48
Female	37
Median age (years)	42 (range, 18–88 years)
Patients treated with preoperative radiation therapy	67 (78.8%)
Patients treated with postoperative radiation therapy	15 (17.6%)
Patients treated with pre- and postoperative radiation therapy	7 (8.2%)
Patients treated with postoperative radiation therapy only	8 (9.4%)
Patients not treated with radiation therapy	10 (11.8%)
Number of patients treated with chemotherapy	39
Location of tumor	
Right thigh	30 (35.3%)
Left thigh	28 (32.9%)
Right lower leg	6 (7.1%)
Left lower leg	4 (4.7%)
Right knee	3 (3.5%)
Buttock	3 (3.5%)
Left knee	2 (2.4%)
Abdomen	2 (2.4%)
Paraspinal	1 (1.2%)
Chest wall	1 (1.2%)
Left shoulder	1 (1.2%)
Back	1 (1.2%)
Vulva	1 (1.2%)
Head and neck	1 (1.2%)
Tumor size	
Mean (cm)	12.0
Median (cm)	10.0
Range (cm)	1.3–35
Median preoperative radiation dose (cGy)	5000 (range, 2000–7100)
Median postoperative radiation dose (cGy)	1600 (range, 1000–7020)
Median postoperative dose in patients receiving preoperative radiation therapy (cGy)	1600 (range, 1000–2200)
Median postoperative dose in patients not receiving preoperative radiation therapy (cGy)	6150 (range, 5940–7020)
Margin status (number of patients)	
Gross positive	2 (2.4%)
Microscopically positive	12 (14.1%)
Close (<1 mm)	50 (58.8%)
Negative	19 (22.3%)
Margin status not reported	2 (2.4%)
Local failures	4/85 (4.7%)
Local failures (patients treated with preoperative radiation therapy)	2/67 (3.0%)
Local failures (patients not treated with preoperative therapy)	2/18 (11%)

**Table 2:** Mean tumor response (patients treated with radiation therapy).

Pre-op RT	77.6%
No pre-op RT	0%
Concurrent chemotherapy	84.7% (95% CI: 75.9–93.4)
No concurrent chemotherapy	69.5%(95% CI: 55.1–83.8)
	*p*=0.061

**Table 3:** Linear regression for tumor response (all patients).

Parameter	Linear coefficient ± SE	*p* value
Use of chemotherapy	24.6 ± 9.4	0.011
Margin status	9.5 ± 9.3	0.311
Tumor size	0.9 ± 0.7	0.209

**Table 4:** Local control (all patients).

Parameter	Hazard ratio	*p* value
Tumor size	HR 1.17 (per cm) (95% CI: 1.02–1.33)	0.022
Margin	HR 0.32 (95% CI: 0.02–4.49)	0.396

**Table 5:** Factors associated with disease-free survival on multivariate analysis (patients treated with radiation therapy).

Parameter	Hazard ratio	*p* value
Necrosis/tumor response	0.90 (per 10% of necrosis) (95% CI: 0.75–1.07)	0.222
High-grade disease	5.97 (95% CI: 0.72–49.67)	0.172
Tumor size	1.08 (per cm) (95% CI: 1.01–1.16)	0.019

**Table 6:** Factors associated with disease-free survival on multivariate analysis.

Parameter	Hazard ratio	*p* value
Use of radiation therapy	HR 0.45 (95% CI: 0.13–1.60)	0.218
Size > 10 cm + grade ≥ 2	HR 3.11 (95% CI: 1.17–8.27)	0.023

**Table 7:** Factors associated with overall survival multivariate analysis.

Parameter	Hazard ratio	*p* value
Any radiation	HR 0.28 (95% CI: 0.06–1.47)	0.133
Size > 10 cm + grade ≥ 2	HR 6.56 (95% CI: 1.32–32.76)	0.022

## Data Availability

The data used to support the findings of this study are available from the corresponding author upon request.
